# Relationship between Vegetation Habitats and Bird Communities in Urban Mountain Parks

**DOI:** 10.3390/ani12182470

**Published:** 2022-09-19

**Authors:** Weizhen Xu, Jiao Yu, Peilin Huang, Dulai Zheng, Yuxin Lin, Ziluo Huang, Yujie Zhao, Jiaying Dong, Zhipeng Zhu, Weicong Fu

**Affiliations:** 1 College of Landscape Architecture, Fujian Agriculture and Forestry University, 15 Shangxiadian Rd, Fuzhou 350002, China; 2 School of Architecture, Clemson University, 105 Sikes Hall, Clemson, SC 29634, USA; 3 College of Architecture and Urban Planning, Fujian University of Technology, 33 Xuefunan Rd, Fuzhou 350118, China; 4 Engineering Research Center for Forest Park of National Forestry and Grassland Administration, 63 Xiyuangong Rd, Fuzhou 350002, China

**Keywords:** bird diversity, spatial and temporal variation, urban mountain parks, vegetation habitats

## Abstract

**Simple Summary:**

The complexity of urban green space vegetation is an extremely vital influential factor and predictor of biodiversity, and vegetation habitat factors have been shown to explain more variation in bird diversity than other environmental factors. In this study, we explored the characteristics and differences in bird diversity among vegetation habitats in five typical urban mountain parks in coastal areas of southeast China. Our study showed that: (1) Sparse forest supports more bird species and higher bird diversity. (2) Tree diversity significantly affects bird diversity in urban mountain parks. (3) The average subbranch height of trees has a significant effect on the evenness of birds. (4) The number of tourists and predators has little impact on bird diversity. This study could provide suggestions for the rational allocation of vegetation in urban mountain parks in southeast coastal areas to improve local ecological service functions as habitats for urban wildlife, especially wild birds.

**Abstract:**

The coastal areas of southeast China have typical high-density urbanization characteristics, and urban mountain parks are important green spaces in these urban green space systems. Our study was conducted in five typical urban mountain parks in Fuzhou, China. The bird survey was carried out in 25 transects of different vegetation habitats for 10 months, and the vegetation survey was conducted in 25 habitats to investigate the characteristics of bird communities in different vegetation habitats and the causes of their differences. The results showed: (1) From 1 October 2021 to 15 July 2022, we recorded a total of 90 bird species in 8 orders, 37 families, and 64 genera, with 1879 individuals in five vegetation habitats in the urban mountain parks. (2) Abundance and diversity of trees are vegetation variables affecting bird diversity (bird species richness, abundance, and Shannon diversity) in urban mountain parks, and the average branch height under trees has a significant effect on bird evenness. (3) We found more bird species and higher bird diversity in both sparse and dense forest habitats, but fewer bird species in waterfront, shrub, and grassland habitats; (4) Average tree height (AVE_HEIt) was only present in the best model of bird abundance and evenness. However, none of the best models found a significant effect of the number of tourists and predators on bird diversity. Our results could provide a theoretical basis and guidance for the future improvement of ecological service functions of bird habitats in urban mountain parks in subtropical coastal areas.

## 1. Introduction

With the continuous acceleration of urbanization, urban ecosystems are seriously damaged [1,2,3]. In the process of creating a harmonious landscape between humans and nature, the level of regional biodiversity continues to decline, and how to effectively maintain biodiversity is one of the greatest challenges for environmental protection in the 21st century [4]. Bird species, as one of the critical components of biodiversity and important elements in different trophic levels of the food chain, are vital indicators of the urban ecosystem’s health [5]. Urban green space [6,7] plays a crucial role in providing suitable habitats for urban wildlife, and plants, as the physical environment on which most wildlife depends, are an essential element in maintaining the balance of urban ecosystems themselves and protecting biodiversity.

Bird species are not only an important indicator to evaluate the quality of the ecological environment in a certain area but are also considered one of the best objects to study urban biodiversity [8,9]. In recent years, studies on the relationship between bird diversity and vegetation have mostly been conducted in natural environments [10,11,12]. However, with the rapid development and spread of urbanization, it is particularly important to conduct such research in urban areas [13,14,15]. The complexity of urban green space vegetation is an extremely vital influence and predictor of biodiversity, and vegetation habitat factors have been shown to explain more variation in bird diversity than other environmental factors [16,17]. Previous studies have also shown that changes in vegetation habitat composition can indirectly or directly alter bird composition and that increasing vegetation structural complexity and species diversity can effectively increase bird diversity [18,19,20]. In urban vegetation communities, the canopy cover of tree forests is reduced compared to the natural environment, and the reduction in tree cover has a negative effect on bird communities [21]. Therefore, the study of urban bird habitats can focus on a certain type of urban green space to provide more effective planning guidelines for decision-makers, and then provide references for future urban green space biodiversity enhancement.

Fuzhou has a richness of bird resources, with 455 species recorded in the China Birding Record Center (http://www.birdreport.cn/, accessed on 13 August 2022) as of August 2022. Therefore, the conservation of wild bird resources in Fuzhou is significant not only for the improvement of local biodiversity but also for the protection of all wild bird species along their migratory routes through Fuzhou [22]. Unlike ordinary urban parks, urban mountain parks are usually built on the unique environment of special topography and natural ecology and are a major part of the urban green space system [23]. Moreover, urban mountain parks exist in a large number of subtropical coastal areas in China, so they are typical and have certain research significance [24]. However, the vegetation configuration of urban mountain parks has been mainly considered for landscape and recreation needs, and its ecological service function as a wildlife habitat has not received sufficient attention [25]. Therefore, studying the relationship between bird communities and vegetation habitats in urban mountain parks is a crucial basic study that can effectively improve the level of bird diversity in urbanized areas.

In this study, we explored the characteristics and differences in bird diversity among vegetation habitats in five typical urban mountain parks in coastal areas of southeast China, intending to address the following four questions: (1) Does bird diversity differ among vegetation habitats in subtropical coastal urban mountain parks? What are the causes of such differences? (2) Does bird diversity vary seasonally in different vegetation habitats? (3) Which vegetation habitat factors affect bird diversity in urban mountain parks? What is the importance of these habitat factors for bird diversity? (4) How can the vegetation habitat of urban mountain parks be reasonably created to enhance bird diversity? In this context, we intend to provide a theoretical basis and suggestions for the rational allocation of vegetation in urban mountain parks in southeast coastal areas to improve their ecological service functions as habitats for urban wildlife, especially wild birds, by answering these questions.

## 2. Study Area and Methods

### 2.1. Study Area

**Figure 1:**
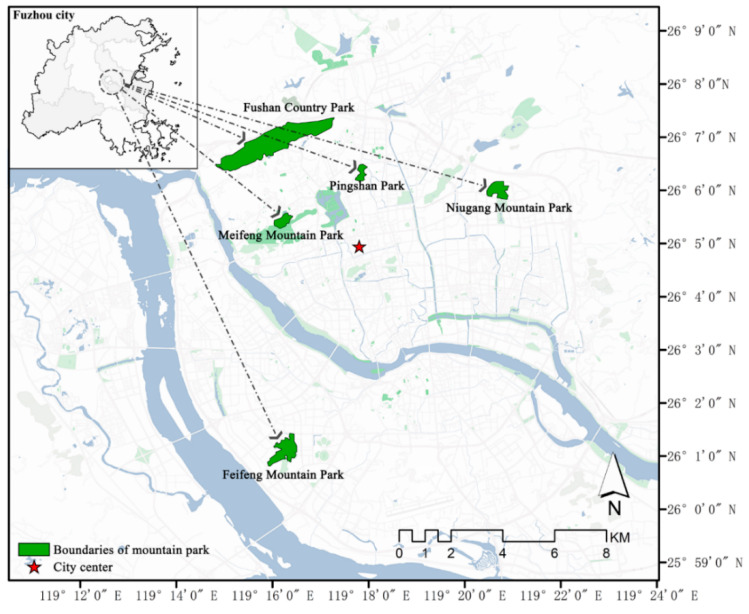
The location map of five urban mountain parks in the subtropical coastal areas of China was selected in this study.

Fuzhou, a typical coastal city in southeast China, was selected as the study area, with latitude and longitude ranging from 25°15′ N~26°39′ N and 118°08′ E~120°31′ E. It is a mountainous city surrounded by many mountains, and there are 58 mountain ranges of different scales in its area [26]. In addition, the Fuzhou Municipal Government has built a number of mountain parks for tourists and citizens to enjoy, all of which are based on the original natural mountain terrain [27]. Therefore, Fuzhou is a representative city for studying bird diversity in mountain parks. According to the urban planning and vegetation situation in Fuzhou and regarding the previous selection methods, the urban mountain parks were systematically sampled according to the following requirements: (1) different and representative locations, and within the main urban area of Fuzhou, i.e., within the Third Ring Expressway; (2) high green area and vegetation coverage, diverse plant composition, and typical habitats; and (3) rich bird resources, which are the main places for surrounding residents or visitors to watch birds. The following five urban mountain parks were selected for the study: Fushan Country Park (235.59 ha, 4~143 m a.s.l.), Meifeng Mountain Park (18.44 ha, 24~103 m a.s.l.), Niugang Mountain Park (29.89 ha, 11~60 m a.s.l.), Feifeng Mountain Park (39.41 ha, 5~62 m a.s.l.), and Pingshan Park (11.86 ha, 12~39 m a.s.l.) (Figure 1). Moreover, all the five urban mountain parks have similar vegetation structures.

### 2.2. Vegetation Habitats and Sampling Criteria

**Figure 2:**
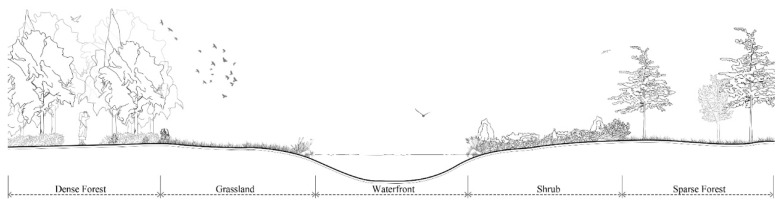
Schematic diagram of five typical vegetation habitats in urban mountain parks. From left to right are dense forest habitat, grassland habitat, waterfront habitat, shrub habitat, and sparse forest habitat.

We conducted a pre-survey of vegetation habitats in the selected five urban mountain parks between 10 and 20 October 2021. Based on the pre-survey, and referring to Ossola (2016) et al. [28] and Yang (2015) et al. [29], a total of five categories of urban mountain park vegetation habitats were classified as follows (Figure 2):

(1) Sparse forest: 30% ≤ tree canopy cover ≤ 70%, mainly tree-turf or trees; (2) dense forest: tree canopy cover > 70%, mainly tree-shrub-ground cover or tree-ground cover; (3) waterfront: lakes, ponds, streams, ditches, etc., vegetation mostly reeds, irises, and other aquatic plants; (4) grassland: tree canopy cover < 30%, mainly herbaceous plants or grassland space; and (5) shrub: tree canopy cover < 30%, mainly shrub-ground cover or shrub. The above five habitats were all present in the five selected urban mountain parks.

Transects [30] of 100 m × 50 m are randomly laid along the internal road of the mountain park as the basic unit of bird survey, with 100 m being the length of the transects and 25 m being the radius within which individuals of each species were counted [31]. The shortest distance between transects should be longer than 100 m to provide spatial independence, and the locations should be scattered as evenly as possible. To ensure scientific and fair results, we used “systematic sampling [32]” to select a sample section containing the above five vegetation habitats in each urban mountain park, and finally, a total of 5 × 5 vegetation habitats were selected for subsequent analysis.

### 2.3. Vegetation Habitat Variables

A standard 20 m × 20 m sample square [33,34] was set in the middle of each transect as the basic unit of the plant survey (randomly sampled along the side of the road toward the interior of the plant community). From May to July 2022, the vegetation survey was carried out in all sample squares using “five-point sampling [35]”. A large sample square of 20 m × 20 m was set up with the survey sample point as the center, and the species and number of tree layers were counted. Within the 20 m × 20 m sample square, five 4 m × 4 m medium sample squares and five 1 m × 1 m small sample squares were randomly selected. The species and number of shrub layers were investigated in the middle sample square, and the species of the ground cover layer were investigated in the small one. The stratification structure of vegetation [36] was divided according to the height of above-ground parts of plants (tree layer, h > 2.5 m; shrub layer, 0.5 m ≤ h ≤ 2.5 m; ground cover layer, h < 0.5 m), and information on 14 vegetation variables (Table S1) was collected as follows.

(1) Diversity of tree layer (DIVt), the diversity index of tree layer within the large sampling square, diversity index was calculated using the Shannon–Wiener index with the formula:
  *H′* = −Σ*P_i_lnP_i_* (1)
  *P_i_* is the ratio of the number of individuals of plant species *i* to the total number of individuals in the community, *i* = 1, 2, …, *S*. *S* is the total number of plant species.
(2) Diversity of shrub layer (DIVs), the diversity index of shrub layer plants within the medium sampling square, calculated by the same formula as the tree layer diversity index.
(3) Richness of tree layer (RICt), the number of species in a tree layer within the large sampling square.
(4) Richness of shrub layer (RICs), the number of species of shrub layer plants in the middle sampling square.
(5) Richness of ground cover (RICg), the number of species of ground cover plants in the small sampling square.
(6) Coverage of tree layer (COVt), the percentage of canopy area of a tree layer to the total area of a large sampling square.
(7) Coverage of shrub layer (COVs), the percentage of shrub layer cover area to medium sampling square.
(8) Coverage of ground cover (COVg), the percentage of ground cover area to a small sampling square.
(9) Abundance of trees (ABUt), the number of individuals in the tree layer in the large sampling square.
(10) Maximum tree height (MAX_HEIt), the maximum tree height of the tree layer in the large sampling square.
(11) Average branch height under trees (AVE_HBt), the average branch height under trees in the tree layer within the large sampling square.
(12) Average shrub heights (AVE_HEIs), the average height of shrub layer plants within the medium sampling square.
(13) Average tree diameter at breast height (AVE_DBH), the average diameter at breast height of plants in the tree layer within the large sampling square.
(14) Average tree height (AVE_HEIt), the average height of plants in the tree layer within the large sampling square.

In addition, we also investigated two environmental variables [37,38] that may significantly affect bird communities:

(15) Number of predators (NoP), the average number of predators (cats, dogs, and other animals that may prey on birds) within the transects during the survey period.
(16) Number of tourists (NoT), the average number of tourists within the transects during the survey.

### 2.4. Bird Survey

A total of four observers participated in this study, all of whom have a specialized ecological background and have been members of the Fujian Bird Watching Society for more than one year. “*A field guide to the birds of China* [39]” was used to identify bird species. Further identification is carried out by photographing or recording the case when bird species cannot be identified at the time. A fixed number of two observers was used for each observation to minimize the influence of personnel changes during the bird survey. Line transect methods [40] were used, and transects were selected at random at the beginning of each survey to control the randomness of observation time. Each survey lasted 10–20 min per transect and ended until no new species were present. The official bird survey period was from 1 October 2021 to 15 July 2022, and the survey frequency was once every three months. Bird surveys were performed once every three months for one day. According to the classification of the four seasons in Fuzhou by the Fujian Meteorological Bureau (http://fj.cma.gov.cn/fzsqxj/, accessed on 10 October 2021), the bird survey in each season is as follows: spring (April 2022), summer (July 2022), autumn (October 2021), and winter (January 2022) to enter the subsequent analysis.

In addition, we classified the behavior of birds [41] among different vegetation habitats into the following three categories:

(1) Perching: birds were observed in resting, calling, preening, singing, etc.
(2) Moving: birds were observed moving among different places, chasing, searching, fighting, etc.
(3) Feeding: birds were observed pecking for food or eating worms, plants, or other food resources.

### 2.5. Analysis

#### 2.5.1. Bird Diversity

Species richness represents the number of all species surveyed in each habitat, measured using the Chao1 estimator [42], which is the lower limit of undetected species richness, calculated as the number of singletons and doubletons. Abundance is calculated as the total number of individuals of all species surveyed in each habitat. The Pielou index [43] is measured using the interspecific encounter probability, which controls for sampling effort and bird species density and uses repeated resampling of data to calculate the probability that two randomly sampled individuals in a combination represent two different species. Bird diversity is measured by the Shannon diversity [44], which is based on the frequency of species and does not favor rare or common species. All four indexes (species richness, abundance, Pielou, and Shannon diversity) were performed in the “vegan” package [45] of R 4.0.2. We then used integrity curves estimated from sample coverage to assess the completeness of sampling. The total species richness of the overall bird community was estimated and compared with individual-based interpolation and extrapolation of abundance data to a multi-indicator model in the “iNEXT” package [46] which takes into account the undetectability of rare species. Further, we checked the Gaussian distribution of the four indexes, and a log10 transformation was performed when indexes did not conform to the normal distribution [47]. Because the shortest distances between transects were all longer than 100 m, there may not be any serious spatial autocorrelation. Finally, we used the Mann–Whitney–Wilcoxon test [48] to compare whether bird diversity differed among vegetation habitats.

#### 2.5.2. Bird Behavior

When birds were observed to occur directly in vegetation within 25 m of the transect, we counted the frequency of each behavior. The frequencies of each bird’s behavior in the five vegetation types were compared using a chi-squared test [49]. Bird species that fly by or are identified by sounds were excluded from behavior analysis because no direct vegetation layer information was collected in these cases.

#### 2.5.3. Non-Metric Multidimensional Scaling

Variation in species composition was measured by species variability. It is determined from the log(X + 1) transformed Bray–Curtis similarity matrix of the abundance data. Its equation is as follows.

(2) $${\mathrm{BC}}_{\mathrm{ij}}=1\;-\;\frac{{2C}_{\mathrm{ij}}}{S_{i}{+S}_{j}}$$

where *C_ij_* is the sum of the minimum values for bird species common to both *i* and *j* only. *S_i_* and *S_i_* are the total numbers of bird species in both *i* and *j*.

To investigate species variability among different vegetation habitats, nonmetric multidimensional scaling (NMDS) [50] was used to analyze the composition of bird communities in different vegetation habitats. Then, we derived the species composition of bird communities between habitats and we compared them using analysis of similarity (ANOSIM), a nonparametric multidimensional ranking method for detecting differences between sample groups of communities, using the function “anosim” from the vegan package of R 4.0.2. ANOSIM generates R statistics [45] that range from 0 to 1 and give an absolute measure of the degree of separation of the groups (R > 0.75, well separated; 0.25 < R < 0.75, overlapping to some extent but different; R < 0.25, almost impossible to separate).

#### 2.5.4. Response of Bird Communities to Different Vegetation Habitats

Fourteen vegetation variables from each vegetation habitat sample were subjected to principal component analysis (PCA) [51] and varimax normalized rotation procedures to facilitate interpretation. PCA reduced the dimensionality of the 14 variables by transforming them into three principal components (the first four principal components explained more than 60% of the total variance). We identified vegetation variables with absolute loading values greater than 0.4 in the four principal components and processed them using Pearson correlation [52] to assess their independence. PCA was calculated using the “prcomp” function of R software. The four PC values and NoP and NoT were then defined as six predictor variables.

These six factors were examined using multiple linear regression models to identify factors that had significant effects on the bird community. The selection of the best model by stepwise regression was based on an information-theoretic approach using the Akaike information criterion [53]. Models that differed from the best model by less than 2 AICc units (ΔAICc ≤ 2) were selected as the most parsimonious models. Stepwise regression was performed in the R software “MuMIn” package. In addition, we used regression trees to assess how vegetation variables changed bird diversity. Regression trees [54] allow hierarchically analyzing the effects of variables using a tree diagram, thus avoiding the problem of multicollinearity between variables. The variables and their tendency to influence the dependent variables were observed by tracking the presence of variables at each branch node. Regression tree analysis was performed using the “rpart” package [55].

All statistical analyses were performed in R 4.0.2.

## 3. Results

### 3.1. Overview

From 1 October 2021 to 15 July 2022, we recorded a total of 90 bird species in eight orders, 37 families, and 64 genera, with 1879 individuals in five vegetation habitats in the urban mountain parks (Table S2). Among them, the five most common bird species were Japanese White-eyes (*Zosterops japonicus)* (243 individuals), Chinese Bulbuls (*Pycnonotus sinensis)* (187 individuals), Tree Sparrows (*Passer montanus)* (187 individuals), Black-throated Tits (*Aegithalos concinnus*) (102 individuals), and Red-whiskered Bulbuls (*Pycnonotus jocosus)* (95 individuals). In terms of residence type, resident bird species occupied the largest proportion (66.67%, 0), followed by winter visitors (16.67%, *n* = 15), summer visitors (11.11%, *n* = 10) and passing migrants (5.56%, *n* = 5). For the IUCN Red List of Threatened Species, LC (least concern) species occupied a larger proportion (97.78%, *n* = 88). One VU (vulnerable) species, Collared Crow (*Corvus torquatus)*, was recorded in Fushan Country Park, and one NT (near threatened) species, Japanese Paradise Flycatcher (*Terpsiphone atrocaudata)*, was recorded in Pingshan Park.

**Figure 3:**
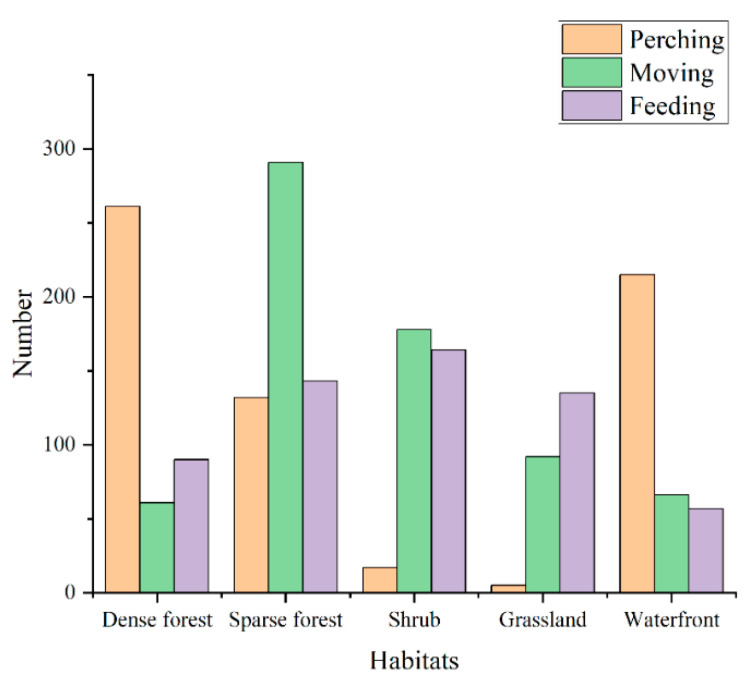
Distribution of different behavioral types of birds in different vegetation habitats in urban mountain parks.

**Figure 4:**
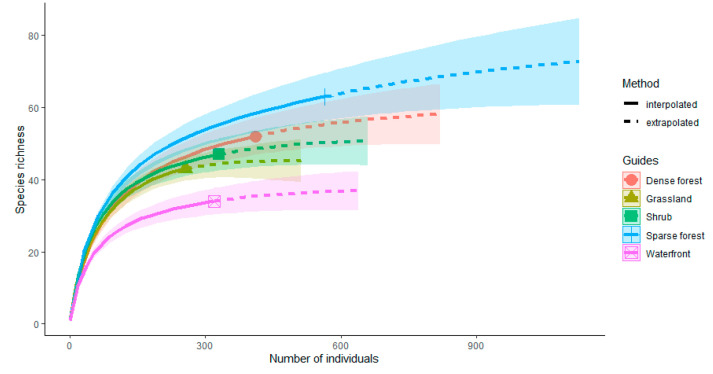
Comparison of individual-based interpolation (sparsity) and extrapolation of bird communities in different vegetation habitats in urban mountain parks under a multi-indicator model.

**Figure 5:**
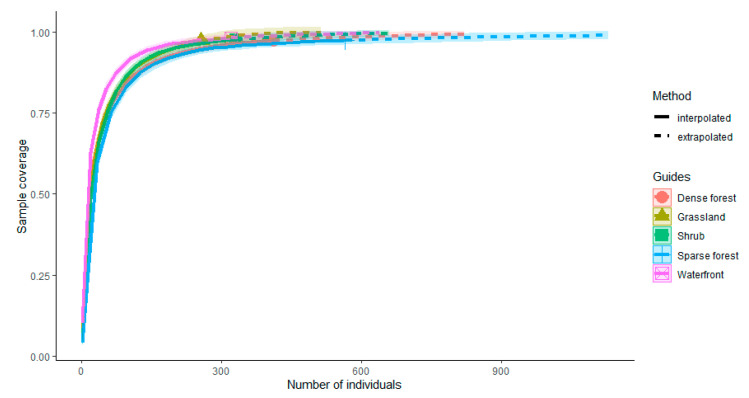
Sample completeness curves of bird communities in different vegetation habitats in urban mountain parks. The filled graphs (triangles, circles, etc.) represent the sample coverage.

We found that 41.43% (261/630) of the perching behavior was observed in the sparse forest (Figure 3). The chi-square test showed that the number of perching behaviors of birds in the sparse forest was significantly different from other habitats (X^2^ = 418.29, df = 4, *p* < 0.001). In terms of moving behaviors, 42.30% of the behaviors occurred in dense forest (291/688). Birds in sparse forest (8.87%, 61/688) and waterfront habitats (9.59%, 66/688) had a similar frequency of moving behaviors. Moreover, the dense forest was highly significantly different from the other habitats (X^2^ = 277.89, df = 4, *p* < 0.001). Similarly, the chi-square test showed that in terms of feeding behavior, the shrub habitat (27.84%, 164/589) had the most feeding behavior and was significantly different from other habitats (X^2^ = 63.963, df = 4, *p* < 0.001), while the water habitat had the least feeding behavior (9.68%, 57/589). The sparsity curve (Figure 4) showed a gradual flattening curve with an increasing number of individuals. Additional bird individuals made a small marginal contribution to the discovery of new species, indicating a gradual saturation of bird species. The sampling completeness of bird surveys in urban mountain parks was greater than 85% (Figure 5), indicating that the entire data set is reasonable and meets the need for further analysis.

### 3.2. Spatial and Temporal Variation of Bird Communities in Different Habitats

#### 3.2.1. Spatial Variation of Bird Communities in Different Habitats

**Figure 6:**
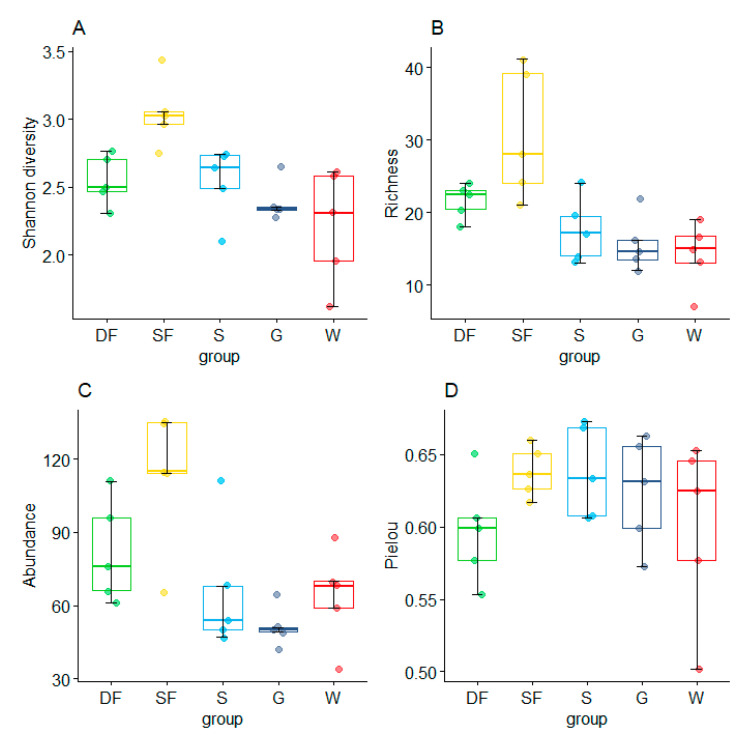
Comparison of bird diversity (**A**), richness (**B**), abundance (**C**), and Pielou index (**D**) in five vegetation habitats in urban mountain parks in Fuzhou, China. DF, dense forest; SF, sparse forest; S, shrub; G, grassland; W, Waterfront.

Among the five different vegetation habitats in the urban mountain park, the sparse forest habitat had the highest Shannon diversity (3.556, mean ± SD = 3.05 ± 0.22), while the water habitat had the lowest Shannon diversity (2.818, mean ± SD = 2.22 ± 0.38) (Figure 6A). The results of the Wilcoxon paired test showed that there were significant differences in Shannon diversity between sparse forest and dense forest (W = 8, *p* = 0.016). The ranking of species richness was sparse forest (*n* = 76, mean ± SD = 30.67 ± 8.07) > dense habitat (*n* = 58, mean ± SD = 21.59 ± 2.12) > shrub (*n* = 50, mean ± SD = 17.53 ± 3.97) > grassland (*n* = 44, mean ± SD = 15.65 ± 3.45) > waterfront (*n* = 37, mean ± SD = 14.15 ± 4.09) (Figure 6B). We also found significant differences between sparse forest and grassland (W = 1, *p* = 0.016), shrub (W = 1.5, *p* = 0.028) and waterfront (W = 25, *p* = 0.008) habitats in species richness. In terms of the number of bird individuals (Figure 6C), sparse forest had the highest number of individuals (*n* = 564, mean ± SD = 112.80 ± 25.60), followed by dense forest (*n* = 410, mean ± SD = 82 ± 18.81), while grasslands had the lowest number of individuals (*n* = 256, mean ± SD = 51.20 ± 7.14). Regarding the Pielou index (Figure 6D), shrub habitat (Pielou index = 0.614, mean ± SD = 0.64 ± 0.03), sparse forest (Pielou index = 0.595, mean ± SD = 0.64 ± 0.16), and grassland (Pielou index = 0.598, mean ± SD = 0.62 ± 0.03) all had high evenness, but waterfront (Pielou index = 0.554, mean ± SD = 0.60 ± 0.06) maintained a lower evenness.

**Figure 7:**
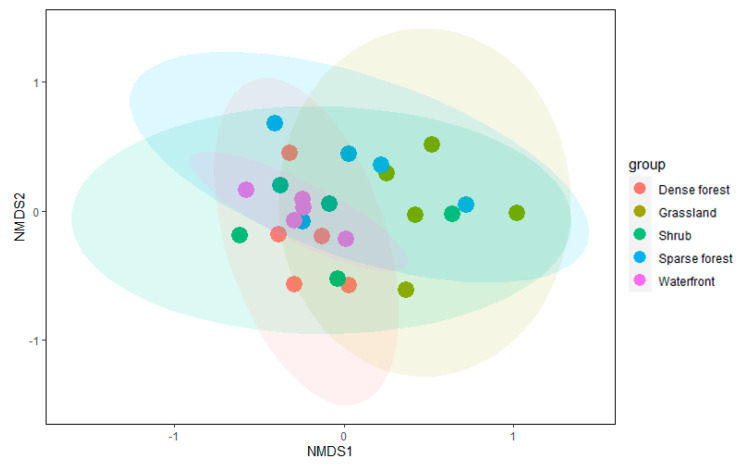
Differences in bird community composition for 25 sampled transects (five dense forest habitats = red circles, five sparse forest habitats = blue circles, five grassland habitats = brown circles, five shrub habitats = green circles, and five waterfront habitats = purple circles) in five different vegetation habitats in urban mountain parks.

The Bray–Curtis function calculated the distances between overall bird communities of five vegetation habitats and ranked the results. Among them, ANOSIM similarity analysis was applied to non-parametric tests. Surprisingly, the results of 999 permutations showed that there was little difference between the groups of overall birds in different vegetation habitats (Figure 7) (Stress = 0.163, ANOSIM statistic R = 0.085, Significance = 0.121).

#### 3.2.2. Temporal Variation of Bird Communities in Different Habitats

**Figure 8:**
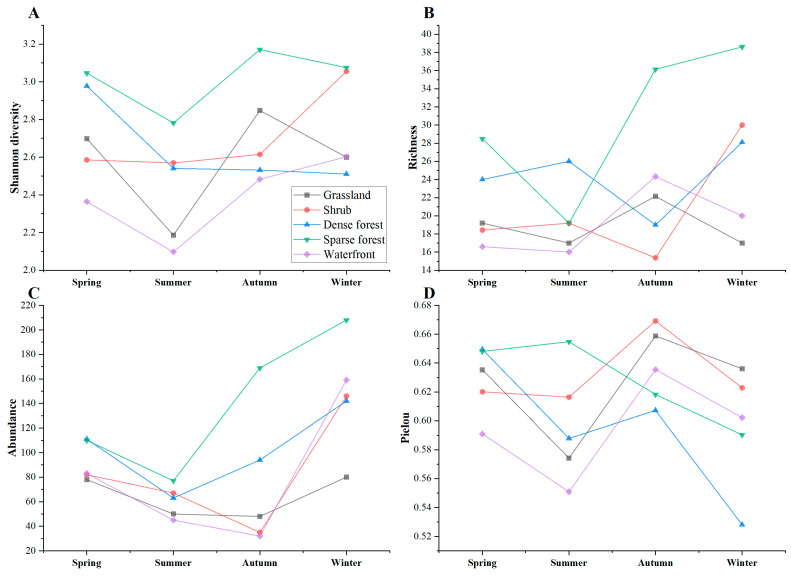
Comparison of bird diversity (**A**), richness (**B**), abundance (**C**), and Pielou index (**D**) with seasonal variation in five habitats in urban mountain parks in Fuzhou, China.

The sparse forest habitat supported higher bird diversity in all seasons (Figure 8A). Moreover, in all habitats, bird diversity was maintained at a lower level in summer. In winter, the shrub habitat (Shannon diversity = 3.056) had about the same level of bird diversity as the sparse forest (Shannon diversity = 3.075). In terms of richness, the number of bird species in sparse forest (richness = 19) decreased significantly in summer and was almost equal to that of shrub habitats (richness = 18). In contrast, bird richness in waterfront and grassland habitats varied similarly throughout the year (Figure 8B). In terms of annual variation in abundance (Figure 8C), the sparse forest maintained a high level of bird abundance throughout the year, and the trend was more consistent with that of dense forest habitat. The number of bird individuals in both habitats decreased to some extent in summer and gradually increased in autumn and winter, reaching a higher value during winter. In terms of annual changes in the Pielou index (Figure 8D), the waterfront, shrub, dense forest, and grassland habitats showed an “N” trend with seasonal migration. In contrast, the Pielou index of sparse forest habitat showed a small increase in summer and a significant decrease in autumn and winter.

### 3.3. Relationship between Bird Diversity and Vegetation Habitats

We used PCA to assess vegetation composition variables for five vegetation habitats in urban mountain parks. The first four components accounted for 62.5% of the total variance (Table 1), so they were selected for further analysis. The variables DIVt, DIVs, and RICt had absolute loading values greater than 0.4 in the first component (PC1, Table 1), and the Pearson correlation test showed a highly significant correlation between DIVt and RICt (correlation = 0.855, *p*-value < 0.001). Thus, PC1 mainly represents the diversity of trees in vegetation habitats. The variables DIVs and AVE_HEIs had absolute loading values greater than 0.4 in PC2, and they symbolize the vegetation diversity in the shrub layer. In the third principal component (PC3), the absolute loading values of COVt, COVg, and AVE_DBH were greater than 0.4, and they mainly represented the shrub cover and the average diameter at breast height of trees in the sample square. In the fourth principal component, only one variable, AVE_HBt, had absolute loading values greater than 0.4, which represented the average height of the branch height of trees in the sample square.

Multiple linear regression models showed that PC1 was a significant factor affecting richness (F-statistic = 4.929, R^2^ = 0.141, *p* = 0.037), abundance (F-statistic = 4.426, R^2^ = 0.222, *p* = 0.024) and Shannon diversity (F statistic = 8.27, R^2^ = 0.265, *p* = 0.009) were essential factors because in these best models of bird diversity (models 1, 4, and 9), they all had ΔAICc < 2 (Table 2). PC4 was only present in the models of abundance (model 4) and Pielou index (model 14), where it had a lower fit (F-statistic = 2.498, R^2^ = 0.059, *p* = 0.013), but also indicated that the average height under tree branches was vital in both bird abundance and evenness. However, neither NoP nor NoT appeared in any of the best models.

**Figure 9:**
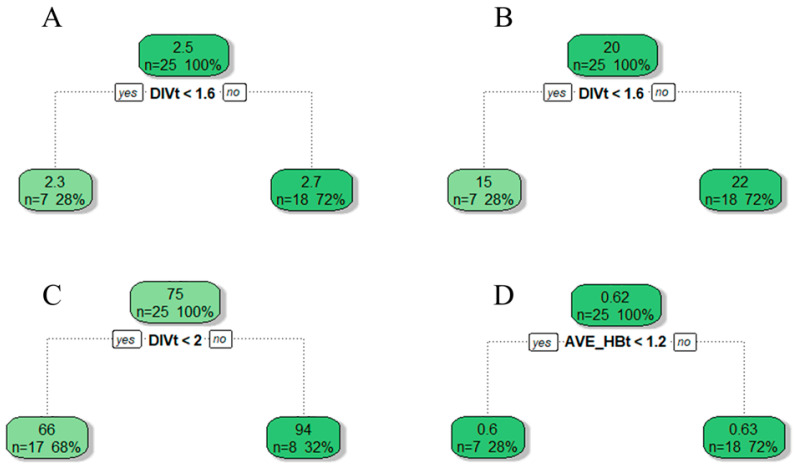
Relationships between bird diversity (**A**), richness (**B**), abundance (**C**), and Pielou index (**D**) of urban mountain parks based on regression tree and vegetation variables from the results of principal component analysis (PC1 to PC4). DIVt, diversity of trees; AVE_HBt, average branch height under trees.

PC1 all appeared as relevant vegetation factors in the best regression models for richness, abundance, and Shannon diversity (Table 2), so the important variables in PC1 (DIVt and RICt) were used to construct regression trees. Since PC4 (AVE_HBt) also appeared in the best regression models for abundance and Pielou, we used PC1 and PC4 to construct regression trees for abundance, but only PC4 was used to construct regression trees in Pielou. For the regression tree of Shannon diversity with PC1 (Figure 9A), DIVt was the main vegetation variable with the most positive effect on Shannon diversity in PC1. And, at DIVt > 1.6, it had a positive effect on bird diversity. Similarly, in the regression tree between richness and PC1 (Figure 9B), DIVt had a positive effect. In addition, at DIVt < 1.6, bird richness decreased. In the regression tree of abundance with PC1 and PC4 (Figure 9C), DIVt was the most dominant vegetation variable with a positive effect, and abundance had a more significant increase when DIVt > 2. In Figure 9D, only AVE_HBt occurred, indicating the average branch height under trees had a more positive effect on the evenness of birds.

## 4. Discussion

### 4.1. Distribution of Bird Communities in Different Vegetation Habitats

Previous studies emphasized that habitat structure type and its compositional complexity were also two critical determinants of bird diversity [16,20,56,57]. Erdelen (1984) [58] found that bird species richness and diversity were often related to vegetation structure rather than just woody species richness. The planning and design of urban mountain parks needs to provide for not only the recreational needs of tourists but also issues such as providing habitats for wildlife [22]. Therefore, various types of vegetation structures have emerged in urban mountain parks, such as dense forests, sparse forests, shrubs, grasslands, and waterfronts as five typical vegetation habitat types, which represent an increase in structural complexity. We recorded more bird species in the sparse forest, dense forest, and shrub habitats, which have a diverse vegetation structure and a more natural land cover than heavily managed vegetation (e.g., artificial grasslands or regular woodlands), giving wild species a better living quality.

### 4.2. Response of Bird Communities to Vegetation Habitat Factors

Tree layers are often considered to be one of the most essential vegetation factors for enhancing bird diversity in urban green spaces because they provide nesting and foraging sites for birds [59]. The regression model of bird diversity and vegetation habitat factors showed that the tree diversity (i.e., PC 1 in Table 1) had a positive effect on bird diversity (Table 2), which indicated that both the structural complexity and diversity of vegetation have a significant positive effect on bird communities in urban mountain parks. Based on regression tree analysis, we highlighted that tree diversity was a critical vegetation variable affecting bird diversity (Figure 8A), richness (Figure 8B), and abundance (Figure 8C). However, in contrast to Fontana et al. (2011) [60] and Yang et al. (2015) [29], we did not find a positive effect of COVt on bird communities, as it was not significant in any of the best regression models. The diversity of the shrub layer was also a vital indicator for the bird community compared to the tree layer [61]. Moreover, the shrub layer had the highest foraging behavior in the results of bird behavior (27.84%, 164/589). Dense shrubs in mountain parks usually reduce the visibility of human visitors, thus reducing human disturbance so that high bird diversity can be maintained in shrub habitats. Waterfront habitats are thought to promote bird activity, and they provide a good food resource in addition to being a daily source for bird species. Therefore, we similarly suggest that the value of water resources for bird communities could be studied in more urban parks based on various types of water system designs.

### 4.3. Temporal and Spatial Changes in Bird Diversity

Birds usually migrate spatially along with the change of seasons [62,63]. As a critical stop station on the East Asian–Australian migratory route of birds, Fuzhou usually undergoes large changes in bird communities during seasonal changes, especially in urban mountain parks. Because mountain parks are covered with natural/semi-natural vegetation and usually have more superior habitats than ordinary urban parks, they become important stopover sites for lots of bird species during migration. Our results showed that bird diversity (Shannon diversity, richness, and abundance) maintained high values in both spring and winter. This may be due to the fact that spring is generally the breeding season for birds and winter is the arrival period for migratory birds, so high bird diversity is maintained during these two periods.

Although Fuzhou is a coastal city [64], it has very high temperatures in summer, which seriously affects the survival and occurrence of bird species, especially on the waterfront in summer, and all four indexes maintained low values in summer. In autumn, a crucial period for vegetation flowering and fruiting as well as feeding behavior of bird species, there was a significant increase in bird diversity in all habitats, especially in sparse forests and dense forests. Interestingly, the Pielou index for shrub, grassland, and waterfront habitats showed similar changes throughout the year. The regression tree results showed that the Pielou index was positively correlated with the average branch height of trees, and in general, a higher average branch height of trees indicated the ability to provide different ecological niches for birds, which, in turn, reduced competition among birds and maintained a higher Pielou index.

### 4.4. Implications for Urban Mountain Parks Management

In urban mountain parks, we found sparse forest supported higher bird diversity. In addition, studies from other regions have identified variables such as the number of tourists and predators as important negative drivers of bird abundance, richness, and diversity [12,17]. However, no significant effects of these two variables on bird diversity were found in our study, which may be attributed to the good ecosystem services of the mountain parks themselves that provide diverse refuge spaces for bird species. Meanwhile, our study found that the compositional diversity of trees had a positive effect on maintaining bird diversity, richness, and abundance, which was also confirmed in previous studies. We found fewer bird species in waterfronts (*n* = 37, mean ± SD = 14.15 ± 4.09) and grasslands (*n* = 44, mean ± SD = 15.65 ± 3.45) than in other habitats, further supporting the role of tree diversity in supporting bird community structure, and urban managers and planners should incorporate these measures into urban green space design and management. The regression tree results showed that the average subbranch height of trees had a positive effect on the aspect of Pielou index enhancement, and it is also one of the key points that should be focused on during the construction of urban mountain parks or other kinds of parks in the future. Moreover, in the construction of mountain parks, in addition to meeting the daily service needs of visitors, diversified and multifunctional vegetation habitats are not only a critical way to enhance biodiversity but also an indispensable part in the process of promoting the synergistic development of humans and nature [65].

## 5. Conclusions

Urban mountain parks are an essential ecological space in the urban green space system of China’s subtropical coastal areas. The construction of these urban mountain parks relies on the original topography, emphasizes the opportunities for natural plant growth, and contains a combination of various types of vegetation. Our study led to the following conclusions:

(1) We found more bird diversity and species in both sparse forest and dense forest habitats. However, fewer species were found in waterfront, shrub, and grassland habitats in different seasons. Moreover, Heyman (2010) [66] confirmed that the removal of 50% of shrubs did not affect local bird diversity, which is useful information for designing management plans for urban green spaces. Previous studies have confirmed that improving bird diversity throughout the city requires enhancing the quality of various types of habitats in urban green spaces, which, in turn, has a contributory effect on bird diversity enhancement.
(2) Both the complexity and structure of vegetation have a significant effect on the bird community in urban mountain parks. Among them, the abundance and diversity of trees are critical vegetation variables affecting bird diversity, while the average branch height under trees has a significant effect on the Pielou index. The diversity and abundance of trees are integral parts of influencing the bird community in urban mountain parks, which facilitates the survival of birds in areas with high levels of human disturbance.
(3) In all regression models, the number of tourists and predators found no significant effect on bird diversity in urban mountain parks. The reason may be that urban mountain parks have good natural vegetation resources, which are the main places for bird species to roost and shelter, and this also emphasizes the maintenance and protection of the existing green space resources in mountain parks. In addition, we hypothesize that bird communities may be greatly influenced by the degree of urbanization or disturbance of other environmental factors.

Therefore, we suggest that further research could be conducted in the future to include more green spaces to examine the integrated relationships between bird communities and other environmental variables. In addition, our study only focused on the direct effects of habitat factors on bird species, while ignoring the indirect effects of interactions within the ecosystem, which is one of the priorities for future research. Our results may provide a theoretical basis and guidance for the future enhancement of the ecological service functions of bird habitats in urban mountain parks in subtropical coastal areas.

## Figures and Tables

**Table 1:** Principal component analysis was performed on the variables (predictor variables) of different vegetation habitats in urban mountain parks.

Variables	PC1	PC2	PC3	PC4
DIVt	0.490	0.108	0.085	0.123
DIVs	0.243	−0.454	0.168	−0.305
RICt	0.465	0.054	0.171	0.246
RICs	0.185	−0.382	−0.295	−0.344
RICg	−0.220	−0.265	0.002	0.180
COVt	0.131	0.115	−0.430	0.209
COVs	0.326	−0.066	−0.287	−0.109
COVg	0.046	0.383	0.420	−0.117
ABUt	0.398	0.008	0.179	0.371
MAX_HEIt	0.294	0.223	−0.351	−0.325
AVE_HBt	0.021	0.349	−0.001	−0.451
AVE_HEIs	0.099	−0.420	0.059	0.154
AVE_DBH	−0.047	0.216	−0.450	0.323
AVE_HEIt	−0.138	−0.068	−0.208	0.192
Standard deviation	1.834	1.453	1.321	1.239
Proportion of Variance	0.240	0.151	0.125	0.110
Cumulative Proportion	0.240	0.391	0.516	0.625

**Table 2:** A multiple linear regression model based on the relationship between simulated bird diversity (predictor variables) and vegetation habitat variables (response variables) under the Akaike information criterion.

NO.	Model	Intercept	df	Loglik	AICc	ΔAICc	*w_i_*
	Richness (Chao1)						
1	PC1	19.917	3	−83.785	174.712	0.000	0.181
2	PC1 + PC2	19.917	4	−83.154	176.309	1.596	0.081
3	NoT + PC1	21.353	4	−83.579	177.157	2.445	0.053
4	PC1 + PC4	19.917	4	−83.743	177.487	2.774	0.045
5	NoP + PC1	20.176	4	−83.764	177.529	2.817	0.044
	Abundance						
4	PC1 + PC4	75.160	4	−115.353	240.706	0.000	0.144
5	PC1	75.160	3	−116.828	240.800	0.093	0.137
6	PC1 + PC2	75.160	4	−116.606	243.213	2.507	0.041
7	NoT + PC1 + PC4	80.976	5	−115.083	243.325	2.619	0.039
8	NoT + PC1	79.901	4	−116.669	243.338	2.632	0.039
	Shannon diversity						
9	PC1	2.549	3	−6.692	20.528	0.000	0.208
10	NoT + PC1	2.649	4	−6.208	22.416	1.888	0.081
11	NoP + PC1	2.594	4	−6.401	22.802	2.274	0.067
12	PC1 + PC4	2.549	4	−6.488	22.975	2.447	0.061
13	PC1 + PC3	2.549	4	−6.506	23.011	2.484	0.060
	Pielou index						
14	PC4	0.619	3	46.284	−85.425	0.020	0.065
15	PC3	0.619	3	45.957	−84.772	0.673	0.047
16	PC3 + PC4	0.619	4	47.355	−84.710	0.735	0.045
17	PC1	0.619	3	45.839	−84.535	0.910	0.041
18	PC1 + PC4	0.619	4	47.223	−84.446	0.999	0.040

## Data Availability

The data used to support the findings of this study are available from the corresponding author upon request.

## Supplementary Materials

The following supporting information can be downloaded at: https://www.mdpi.com/article/10.3390/ani12182470/s1, Table S1: Five vegetation habitats and 11 vegetation variables in urban mountain parks in Fuzhou, China. Table S2: Bird list of urban mountain parks and the locations where the birds were observed in Fuzhou, China.
