# Supplementary material for: Relationship between Vegetation Habitats and Bird Communities in Urban Mountain Parks
Source: Animals (Basel). 2022 Sep 19;12(18):2470. doi: 10.3390/ani12182470 (PMC9495260; doi:10.3390/ani12182470)
Supplement: Supplementary file 1 [file animals-12-02470-s001.zip › animals-1901394-supplementary.pdf]

Table S1. Five vegetation habitats and 11 vegetation variables in urban mountain parks in Fuzhou, China.

Variables	Vegetation habitats				
	**Waterfront**	**Sparse forest**	**Dense forest**	**Shrub**	**Grassland**
DIVt	1.34±0.27	2.30±0.19	2.36±0.13	1.36±0.43	1.30±0.31
DIVs	1.12±0.60	0.80±0.45	1.20±0.27	1.30±0.27	0.52±0.42
RICt	2.80±0.40	4.60±0.63	5.40±0.49	2.80±0.75	2.50±0.49
RICs	2.40±0.80	2.06±0.63	2.12±0.35	2.60±0.49	1.50±0.80
RICg	1.60±0.43	1.43±0.55	1.80±0.32	1.54±0.40	1.67±0.49
COVt	22.97±6.26	53.47±11.73	83.32±4.83	19.49±5.49	18.58±5.53
COVs	26.15±13.41	23.97±4.43	45.39±17.32	67.62±12.90	21.00±9.20
COVg	74.95±20.91	85.25±7.67	77.56±7.30	75.45±10.02	92.85±5.76
ABUt	4.40±0.49	6.00±1.10	9.60±1.80	3.60±0.80	3.20±0.40
MAX_HEIt	6.70±1.02	7.42±0.70	10.74±2.22	5.62±1.11	5.26±0.91
AVE_HBt	2.06±0.89	4.03±0.71	6.50±1.16	2.13±0.43	1.72±0.56
AVE_HEIs	0.68±0.07	0.85±0.26	0.79±0.23	0.73±0.25	0.66±0.16
AVE_DBH	0.24±0.04	0.29±0.03	0.36±0.05	0.33±0.05	0.31±0.04
AVE_HEIt	5.00±1.31	5.41±0.43	8.77±1.25	5.07±0.86	4.33±0.76

(1) DIVt, diversity of tree layer; (2) DIVs, Diversity of shrub layer; (3) RICt, richness of tree layer; (4) RICs, richness of shrub layer; (5) RICg, richness of ground cover; (6) COVt, coverage of tree layer; (7) COVs, coverage of shrub layer; (8) COVg, coverage of ground cover; (9) ABUt, abundance of trees; (10) MAX_HEIt, maximum tree height; (11) AVE_HBt, average branch height under trees; (12) AVE_HEIs, average shrub heights; (13) AVE_DBH, average tree diameter at breast height; (14) AVE_HEIt,a verage tree height;

Table S2. Bird list of urban mountain parks and the locations where the birds were observed in Fuzhou, China. LC, least concern; NT, near threatened; VU, vulnerable; PP, Pingshan Park; FCP, Fushan Country Park; NMP, Niugang Mountain Park; MMP, Meifeng Mountain Park; FMP, Feifeng Mountain Park;

No.	Species name	Scientific name	Order	Family	Genus	Resident type	IUCN Red List of Threatened Species	Observed locations
1	Pied Kingfisher	Ceryle rudis	Coraciiformes	Alcedinidae	Ceryle	resident	LC	FCP
2	White-throated Kingfisher	Halcyon smyrnensis	Coraciiformes	Alcedinidae	Halcyon	resident	LC	FCP
3	Collared Dove	Streptopelia decaocto	Columbiformes	Columbidae	Streptopelia	resident	LC	PP
4	Oriental Turtle Dove	Streptopelia orientalis	Columbiformes	Columbidae	Streptopelia	resident	LC	PP NMP MMP FMP FCP
5	Spotted Dove	Spilopelia chinensis	Columbiformes	Columbidae	Spilopelia	resident	LC	PP NMP MMP FMP FCP
6	Common Moorhen	Gallinula chloropus	Gruiformes	Rallidae	Gallinula	resident	LC	PP NMP FMP
7	White-breasted Waterhen	Amaurornis phoenicurus	Gruiformes	Rallidae	Amaurornis	resident	LC	PP MMP FMP FCP
8	Watercock	Gallicrex cinerea	Gruiformes	Rallidae	Gallicrex	summer visitor	LC	MMP
9	Silver Pheasant	Lophura nycthemera	Galliformes	Phasianidae	Lophura	resident	LC	MMP
10	Chinese Bamboo Partridge	Bambusicola thoracicus	Galliformes	Phasianidae	Bambusicola	resident	LC	MMP
11	Plaintive Cuckoo	Cacomantis merulinus	Cuculiformes	Cuculidae	Cacomantis	summer visitor	LC	PP NMP MMP FMP FCP
12	Cuckoo	Cuculus canorus bakeri	Cuculiformes	Cuculidae	Cuculus	summer visitor	LC	PP FMP FCP
13	Greater Coucal	Centropus sinensis	Cuculiformes	Cuculidae	Centropus	resident	LC	PP MMP FCP

14	Indian Cuckoo	Cuculus micropterus	Cuculiformes	Cuculidae	Cuculus	summer visitor	LC	PP
15	Asian Koel	Eudynamys scolopaceus	Cuculiformes	Cuculidae	Eudynamys	summer visitor	LC	FCP
16	Himalayan Cuckoo	Cuculus saturatus	Cuculiformes	Cuculidae	Cuculus	summer visitor	LC	PP
17	Collared Crow	Corvus torquatus	Passeriformes	Corvidae	Corvus	resident	VU	FCP
18	Japanese Paradise Flycatcher	Terpsiphone atrocaudata	Passeriformes	Monarchidae	Terpsiphone	passing migrant	NT	PP
19	Japanese White-eye	Zosterops japonicus	Passeriformes	Zosteropidae	Zosterops	resident	LC	PP NMP MMP FMP FCP
20	Crested Myna	Acridotheres cristatellus	Passeriformes	Sturnidae	Acridotheres	resident	LC	PP NMP MMP FMP FCP
21	Pale Thrush	Turdus pallidus	Passeriformes	Turdidae	Turdus	winter visitor	LC	FMP FCP
22	Sooty-headed Bulbul	Pycnonotus aurigaster	Passeriformes	Pycnonotidae	Pycnonotus jocosus	resident	LC	PP MMP
23	White Wagtail	Motacilla alba	Passeriformes	Motacillidae	Motacilla	resident	LC	PP MMP FMP FCP
24	White-browed Laughingthrush	Garrulax sannio	Passeriformes	Leiothrichidae	Garrulax	resident	LC	MMP FCP
25	White-spectacled Warbler	Seicercus affinis	Passeriformes	Phylloscopidae	Seicercus	winter visitor	LC	PP
26	Eyebrowed Thrush	Turdus obscurus	Passeriformes	Turdidae	Turdus	passing migrant	LC	FMP FCP
27	Tristram's Bunting	Emberiza tristrami	Passeriformes	Emberiza	Emberiza	winter visitor	LC	PP NMP FMP FCP
28	Chinese Bulbul	Pycnonotus sinensis	Passeriformes	Pycnonotidae	Pycnonotus jocosus	resident	LC	PP NMP MMP FMP FCP
29	White-rumped Munia	Lonchura striata	Passeriformes	Passeridae	Lonchura	resident	LC	FMP
30	Scaly-breasted Munia	Lonchura punctulata	Passeriformes	Passeridae	Lonchura	resident	LC	FCP

31	Daurian Redstart	Phoenicurus auroreus	Passeriformes	Muscicapidae	Phoenicurus	winter visitor	LC	PP NMP FCP
32	Fork-tailed Sunbird	Aethopyga christinae	Passeriformes	Nectariniidae	Aethopyga	resident	LC	FCP
33	Orange-bellied Leafbird	Chloropsis hardwickii	Passeriformes	Chloropseidae	Chloropsis	resident	LC	FCP
34	Scarlet Minivet	Pericrocotus speciosus	Passeriformes	Passeriformes	Pericrocotus	resident	LC	PP FMP FCP
35	Plain Prinia	Prinia inornata	Passeriformes	Cisticolidae	Cisticola	resident	LC	PP NMP MMP FMP FCP
36	Large-billed Crow	Corvus macrorhynchos	Passeriformes	Corvidae	Corvus	resident	LC	NMP
37	Dusky Fulvetta	Alcippe brunnea	Passeriformes	Pellorneidae	Schoeniparus	resident	LC	PP
38	Black Bulbul	Hypsipetes leucocephalus	Passeriformes	Pycnonotidae	Hypsipetes	resident	LC	PP NMP MMP FMP
39	Black Drongo	Dicrurus macrocercus	Passeriformes	Dicruridae	Dicrurus	summer visitor	LC	PP
40	Masked Laughingthrush	Garrulax perspicillatus	Passeriformes	Leiothrichidae	Garrulax	resident	LC	NMP MMP FMP FCP
41	Black-collared Starling	Gracupica nigricollis	Passeriformes	Sturnidae	Sturnus	resident	LC	NMP MMP FCP
42	Chinese Grosbeak	Eophona migratoria	Passeriformes	Fringillidae	Eophona	winter visitor	LC	PP MMP FMP
43	Black-naped Oriole	Oriolus chinensis	Passeriformes	Oriolidae	Oriolus	summer visitor	LC	PP NMP FCP
44	Red-whiskered Bulbul	Pycnonotus jocosus	Passeriformes	Pycnonotidae	Pycnonotus jocosus	resident	LC	PP NMP MMP FMP FCP
45	Siberian Rubythroat	Turdinae	Passeriformes	Muscicapidae	Luscinia	passing migrant	LC	PP
46	Rufous-capped Babbler	Stachyridopsis ruficeps	Passeriformes	Timaliidae	Stachyris	resident	LC	FMP FCP

47	Black-throated Bushtit	Aegithalos concinnus	Passeriformes	Aegithalidae	Aegithalos	resident	LC	PP NMP MMP FMP FCP
48	Orange-flanked Bluetail	Tarsiger cyanurus	Passeriformes	Muscicapidae	Tarsiger	winter visitor	LC	NMP
49	Red-billed Blue Magpie	Urocissa erythroryncha	Passeriformes	Corvidae	Urocissa	resident	LC	NMP MMP FCP
50	Hwamei	Garrulax canorus	Passeriformes	Leiothrichidae	Garrulax	resident	LC	PP NMP MMP FMP FCP
51	Yellow-bellied Prinia	Prinia flaviventris	Passeriformes	Cisticolidae	Cisticola	resident	LC	PP NMP MMP FMP FCP
52	Yellow-bellied Tit	Pardaliparus venustulus	Passeriformes	Paridae	Parus	resident	LC	PP NMP MMP FCP
53	Motacilla flava	Motacilla flava	Passeriformes	Motacillidae	Motacilla	winter visitor	LC	MMP
54	Narcissus Flycatcher	Narcissina Flycatcher	Passeriformes	Muscicapidae	Ficedula	passing migrant	LC	PP MMP FCP
55	Yellow-browed Warbler	Phylloscopus inornatus	Passeriformes	Phylloscopidae	Phylloscopus	winter visitor	LC	PP NMP MMP FMP FCP
56	Pallas's Leaf Warbler	Phylloscopus proregulus	Passeriformes	Phylloscopidae	Phylloscopus	winter visitor	LC	PP NMP FMP FCP
57	Grey-chinned Minivet	Pericrocotus solaris	Passeriformes	Passeriformes	Pericrocotus	resident	LC	PP MMP FMP FCP
58	Grey Wagtail	Motacilla cinerea	Passeriformes	Motacillidae	Motacilla	winter visitor	LC	PP MMP FCP
59	Grey Treepie	Dendrocitta formosae	Passeriformes	Corvidae	Dendrocitta	resident	LC	PP NMP MMP FMP FCP
60	Black-faced Bunting	Emberiza spodocephala	Passeriformes	Emberiza	Emberiza	summer visitor	LC	MMP
61	Common Myna	Acridotheres tristis	Passeriformes	Sturnidae	Acridotheres	resident	LC	NMP MMP FMP FCP
62	Barn Swallow	Hirundo rustica	Passeriformes	Hirundinidae	Hirundo	summer visitor	LC	MMP FMP
63	Red-rumped Swallow	Cecropis daurica	Passeriformes	Hirundinidae	Cecropis	resident	LC	MMP

64	Blue Rock-thrush	Monticola solitarius	Passeriformes	Turdidae	Monticola	winter visitor	LC	FMP
65	Chestnut Bulbul	Hemixos castanonotus	Passeriformes	Pycnonotidae	Hypsipetes	resident	LC	PP NMP MMP FCP
66	Collared Finchbill	Spizixos semitorques	Passeriformes	Pycnonotidae	Spizixos	resident	LC	PP NMP MMP FMP FCP
67	Mountain Bulbul	Ixos mcclellandii	Passeriformes	Pycnonotidae	Hypsipetes	resident	LC	NMP MMP FMP FCP
68	Tree Sparrow	Passer montanus	Passeriformes	Passeridae	Passer	resident	LC	PP NMP MMP FMP FCP
69	Brownish-flanked Bush Warbler	Horornis fortipes	Passeriformes	Cettiidae	Cettia	resident	LC	PP MMP FMP FCP
70	Oriental Magpie Robin	Copsychus saularis	Passeriformes	Muscicapida e	Copsychus	resident	LC	PP NMP MMP FMP FCP
71	Japanese Bush-Warbler	Horornis diphone	Passeriformes	Sylviidae	Cettia	winter visitor	LC	MMP
72	Forest Wagtail	Dendronanthus indicus	Passeriformes	Motacillidae	Dendronanth us	winter visitor	LC	NMP MMP FMP
73	Russet Sparrow	Passer rutilans	Passeriformes	Passeridae	Passer	resident	LC	NMP
74	Olive-backed Pipit	Anthus hodgsoni	Passeriformes	Motacillidae	Anthus	winter visitor	LC	PP NMP MMP FCP
75	Common Blackbird	Turdus merula	Passeriformes	Turdidae	Turdus	resident	LC	PP NMP MMP FMP FCP
76	Oriental Skylark	Alauda gulgula	Passeriformes	Alaudidae	Alauda	resident	LC	NMP
77	Japanese Tit	Parus minor	Passeriformes	Paridae	Parus	resident	LC	PP NMP MMP FMP FCP
78	Manchurian Bush Warbler	Horornis borealis	Passeriformes	Cettiidae	Cettia	winter visitor	LC	PP NMP FMP FCP
79	Common Tailorbird	Orthotomus sutorius	Passeriformes	Sylviidae	Orthotomus	resident	LC	NMP MMP FMP FCP

80	Scarlet-backed Flowerpecker	Dicaeum cruentatum	Passeriformes	Dicaeidae	Dicaeum	resident	LC	FMP
81	European Starling	Sturnus vulgaris	Passeriformes	Sturnidae	Sturnus	passing migrant	LC	FCP
82	Long-tailed Shrike	Lanius schach	Passeriformes	Laniidae	Lanius	resident	LC	PP NMP
83	Vinous-throated Parrotbill	Sinosuthora webbiana	Passeriformes	Sylviidae	Paradoxornis	resident	LC	PP MMP FCP
84	Little Egret	Egretta garzetta	Pelecaniformes	Ardeidae	Egretta	resident	LC	PP NMP FMP
85	Chinese Pond Heron	Ardeola bacchus	Pelecaniformes	Ardeidae	Ardeola	resident	LC	PP
86	Black-crowned Night Heron	Nycticorax nycticorax	Pelecaniformes	Ardeidae	Ardeidae	resident	LC	PP FMP
87	Crested Goshawk	Accipiter trivirgatus	Accipitriformes	Accipitridae	Accipiter	resident	LC	FMP
88	Crested Serpent Eagle	Spilornis cheela	Accipitriformes	Accipitridae	Spilornis	resident	LC	FCP
89	Azure-winged Magpie	Cyanopica cyanus	Passeriformes	Corvidae	Cyanopica	resident	LC	PP FCP
90	Blue Whistling Thrush	Myophonus caeruleus	Passeriformes	Muscicapidae	Myophonus	resident	LC	FCP FCP
